# The Effect of Botulinum Toxin Type A in the Autologous Fat Grafting: A Review

**DOI:** 10.1111/jocd.16550

**Published:** 2024-09-21

**Authors:** Chihchieh Lo, Lideng Cao, Yuanyou Lin, Hang Wang

**Affiliations:** ^1^ Department of Oral and Maxillofacial Surgery and State Key Laboratory of Oral Diseases Sichuan University West China College of Stomatology Chengdu Sichuan China

**Keywords:** adipocyte implantation, angiogenesis, botulinum toxin, fat grafting

## Abstract

**Background:**

Autologous fat grafting is a widely used technique in plastic and reconstructive surgery for soft tissue augmentation. Despite its advantages, the primary limitation is the unpredictable retention rate of transplanted fat. Recent studies suggest that botulinum toxin type A (BTX‐A) can enhance fat graft survival by promoting angiogenesis and muscle paralysis.

**Aims:**

This review explores the potential of BTX‐A as an adjuvant in autologous fat grafting, providing insights into its mechanisms, benefits, and the need for further clinical validation.

**Patients/Methods:**

A literature review was conducted using PubMed, Web of Science, MEDLINE, and Embase. Keywords related to BTX‐A, fat grafting, fat graft survival, and angiogenesis were used. Comparative studies reporting histological changes following BTX‐A application in fat grafting were included. Exclusion criteria involved case reports with fewer than three animals, reviews, and letters.

**Results:**

The initial search yielded 108 articles, with seven experimental studies meeting the criteria. These studies demonstrated that BTX‐A enhances fat graft retention by promoting vascularization and adipose‐derived stem cell differentiation. However, these results are mainly based on small animal models.

**Conclusions:**

While BTX‐A shows promise in improving autologous fat grafting outcomes, its efficacy and safety in humans need validation through large‐scale clinical trials. Translating these preclinical findings into human trials is crucial to establish standardized protocols and optimize clinical outcomes. Future research should focus on optimizing dosage and injection sites, conducting long‐term follow‐up studies, and performing multicenter trials to verify the findings.

## 1 Introduction

Since Neuber's pioneering attempt at autologous fat grafting in 1893 [1], this technique has become an essential tool in plastic surgery and soft tissue reconstruction. In recent years, autologous fat grafting has gained significant popularity in both plastic and reconstructive surgery, serving as a common method for trauma recovery and soft tissue augmentation. When compared to synthetic materials, autologous fat offers numerous advantages, including immune compatibility, cost‐effectiveness, natural sensation, and biocompatibility [2]. As a result, autologous fat grafting has found widespread applications in reconstructive surgery, aesthetics, and regenerative medicine for addressing soft tissue defects [3].

Nevertheless, the primary limitation of autologous fat grafting lies in its uncertain and unpredictable retention rate, as the transplanted fat is inevitably absorbed over time, regardless of the success of the procedure [4]. Even with advanced techniques and the transplantation of large fat volumes, absorption remains a challenge [5, 6]. Therefore, the main objective of autologous fat grafting is to enhance the survival rate of the transplanted adipose tissue.

To improve the retention of adipose tissue, various studies have proposed different approaches in recent years. While operational techniques, such as adipose tissue harvesting, purification, processing, injection, and transplantation, play a crucial role in enhancing the survival rate of adipose tissue, other factors also contribute significantly [7, 8, 9]. One important aspect is to improve the survival and preservation rate of adipose tissue. Aside from minimizing losses during the fat tissue harvesting process, the environment of the recipient area and the various cytokines in adipose tissue that promote adipose tissue growth and differentiation are key factors in improving the survival rate of adipose tissue grafts [10, 11, 12, 13, 14, 15, 16]. Several adjuvant treatments, including epidermal growth factor (EGF), platelet‐rich plasma, fibrin glue, vascular endothelial growth factor (VEGF), and BTX‐A, have shown promise in enhancing preservation rates during autologous fat grafting [17, 18, 19, 20, 21].

### 1.1 Factors Affecting the Survival Rate of Fat Grafting

**FIGURE 1:**
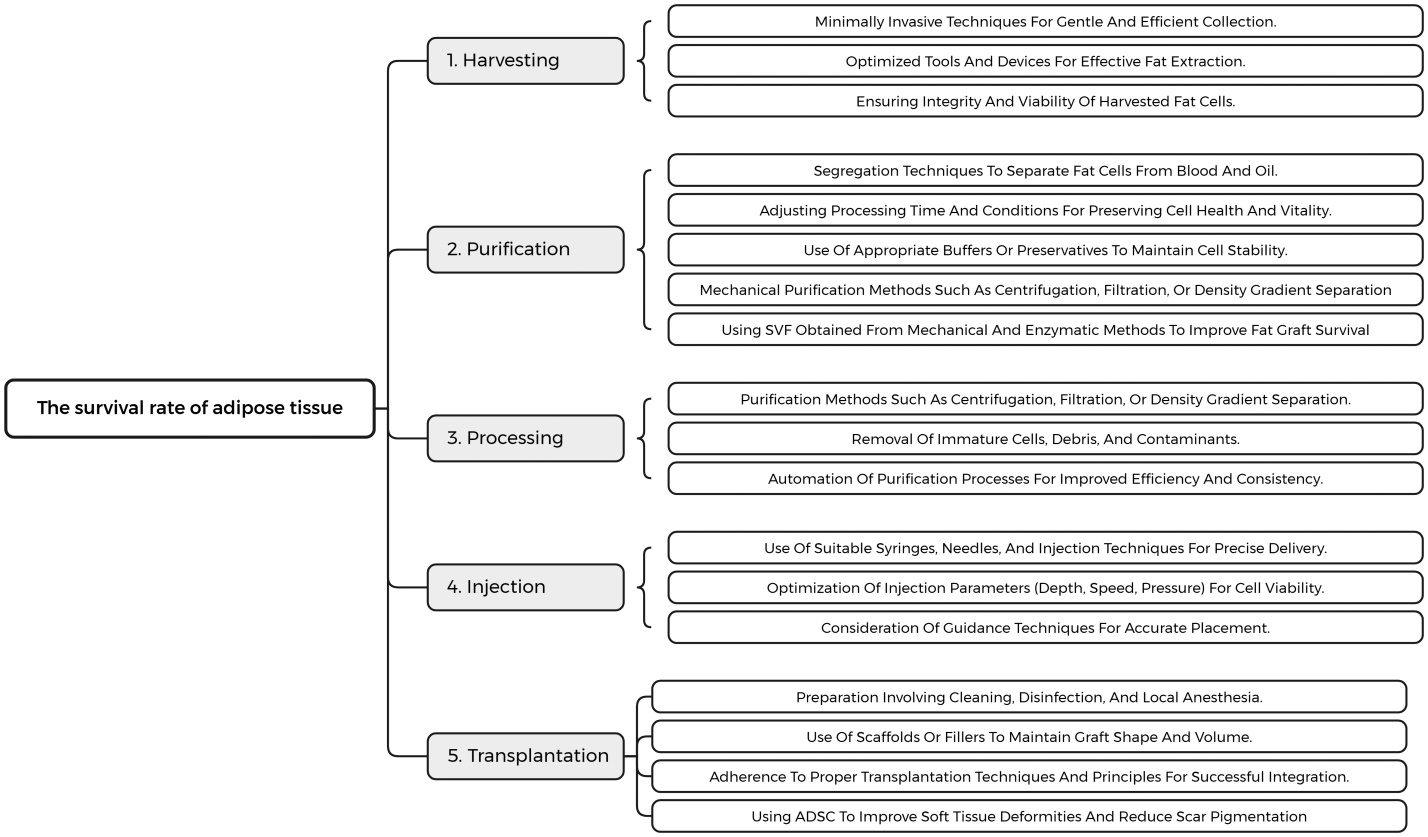
Several effective ways to enhance the survival rate of adipose tissue.

The extraction, processing, purification, injection, and transplantation methods play crucial roles in enhancing the survival rate of adipose graft. Gentle harvesting techniques, optimized processing conditions, and purification steps ensure the integrity and viability of fat cells. Precise injection techniques and appropriate parameters promote cell survival during transplantation. By carefully considering and improving each aspect, the overall success of adipose grafting can be significantly improved, leading to enhanced survival and integration of the transplanted fat cells (Figure 1).

But let us assume that the quality of the collected adipose tissue remains consistent under the same conditions, and the same syringe is used for fat grafting, various factors in the condition of the transplant recipient area can significantly affect the viability of the grafted tissue. In particular, areas with a deficiency of adipose tissue, poor blood flow, malnourished regions, and localized fibroblastic adhesions pose challenges to successful graft integration. Therefore, the establishment of an optimal receiving area for the grafts is crucial. However, most of the areas where adipose tissue is grafted in the body consist of muscle and skin tissue, which may disrupt the growth and preservation of adipose tissue due to shear and stretching forces during movement and exercise. It has been suggested and hypothesized that shear forces can disrupt neovascularization and subsequently impact the volume retention of fat grafts [22]. Compared to subcutaneous grafting, adipose tissue grafted into the muscle is more susceptible to compressive and tensile forces that can disrupt neovascularization in the adipose tissue. Based on this hypothesis, maintaining favorable recipient area conditions can protect neovascularization in adipose tissue from destruction, thus increasing the survival and preservation rate of the grafts.

In addition to the recipient region, the content of growth factors in adipose grafts is a crucial factor in graft survival and retention. Several theories regarding the transplantation of fat grafts have been proposed, including the host replacement theory, the retention theory, and the “three zones” theory [23, 24, 25]. These theories emphasize the importance of angiogenesis and adipose‐derived stem cells (ADSCs) in fat graft retention. Studies have reported that a significant portion of the fat in grafts originates from the differentiation of ADSCs rather than from the autologous fat grafts themselves [24]. Furthermore, ADSCs have been shown to enhance angiogenesis by promoting the formation of new vascular components or secreting angiogenic cytokines [26, 27, 28].

To summarize, to improve the survival and preservation rate of fat grafts, attention should be given to addressing the factors related to the recipient area to prevent mechanical forces from compromising adipose tissue. Additionally, within a stable recipient area environment, increasing the content of growth factors in the adipose tissue can promote angiogenesis and enhance the survival and preservation rate of the grafts. BTX‐A seems to hold promise in addressing these two factors. Apart from its role in inhibiting neurotransmitter transmission to paralyze muscles, there is literature suggesting that BTX‐A possesses unrecognized potential in enhancing angiogenesis.

### 1.2 BTX‐A and Autologous Fat Grafting

BTX‐A, a potent psychotropic toxin produced by the bacterium *Clostridium anaerobium*, exerts its effects by inducing chemical innervation of presynaptic neurons, leading to the inhibition of acetylcholine release and resulting in chemical neurological disorders and muscle dysfunction for up to 6 months [29]. Widely recognized for its applications in the medical aesthetic field, BTX‐A has garnered attention for its potential in various other areas, including facial lifting [30], anti‐photoaging effects on the skin, improving the survival rate of flap grafts [31, 32, 33], scar reduction [34, 35], and notably, enhancing the survival rate of autologous fat grafting [17, 18, 19, 20, 36, 37].

Among the various adjuvant treatments, BTX‐A has emerged as a promising option for improving the survival rate of autologous fat grafting. The inhibitory effect of BTX‐A on muscle activation has been suggested as a potential solution to counteract the impact of muscle movement‐induced shear forces on delicate neovascularization processes, as proposed in a study of skin grafts [22]. Additionally, recent studies have demonstrated that BTX‐A can increase the production of VEGF and promote the differentiation of ADSCs. These findings suggest that BTX‐A may serve as an inducer for adipogenesis, enhancing the concentration of cytokines such as C/EBPα, peroxisome proliferator‐activated receptor (PPARγ), lipocalin, leptin, C/EBPδ, and C/EBPβ, which are known to facilitate ADSC differentiation [18].

Consequently, BTX‐A has shown promising applications in enhancing fat survival and preservation rates, further solidifying its role as a valuable adjuvant treatment in autologous fat grafting.

## 2 Methods

This literature review was undertaken of these major databases: PubMed, Web of Science, MEDLINE, and Embase were searched using a broad range of terms and keywords related to botulinum toxin type A, fat grafting, fat graft survival, and angiogenesis. All comparative studies reporting histology changes following the application of BTX‐A, fat grafting were included. Case reports or series with less than three animals were excluded, as were reviews and letters. One reviewer undertook the searches, and the references of included studies underwent hand searching for other potentially relevant material. There were no restrictions on time or language of publication. The primary outcome was changed in histology following application of fat grafts.

## 3 Results

The initial search returned 108 full‐text articles, 101 of which were excluded after applying the predetermined criteria. Consequently, a total of seven experimental studies published between 2004 and 2024 have been included in this review.

Although the precise mechanism and effects of BTX‐A in the context of fat grafting have not been fully elucidated in the literature, the aforementioned research results clearly demonstrate its potential benefits and applications in autologous fat grafting (Table 1).

**TABLE 1:** Effects of botulinum toxin on fat survival: Author, recipient species, implantation, timing, and assessed outcomes.

Author	Recipient species	Injection dose (U)	Implantation site	Max follow‐up	Outcomes
Baek et al. [17]	Rat	0.5	Back (muscle)	9 weeks	Increased fat survival rate Better cell integrity
Tang et al. [18]	Rat	0.2	Back (muscle)	5 weeks	Increased fat survival rate Better cell integrity Promotion of angiogenesis
Wu et al. [19]	Rat	0.5	Quadriceps femoris	12 weeks	Increased fat survival rate Promotion of angiogenesis
Shi et al. [36]	Rat	2.0	Quadriceps femoris	12 weeks	Higher density of vessels Better cell integrity More mature adipocytes Less fibrosis
Shi et al. [20]	Rat	2.0	Subcutaneous space	12 weeks	Higher density of vessels Better cell integrity More mature adipocytes
Yoon et al. [21]	Rabbit	1.0	Ears	10 weeks	Increased fat survival rate Higher density of vessels Less fibrosis
Jung et al. [37]	Murine		Subcutaneous space	6 weeks	Increased fat survival rate Promotion of angiogenesis

### 3.1 BTX‐A Increases Graft Preservation by Paralyzing Muscle Movement

In 1998, it was hypothesized that shear forces could disrupt neovascularization and negatively impact the volume retention of fat grafts [22]. However, this hypothesis was primarily based on fat grafts injected into the skin. In autologous fat grafting, the grafts are often injected not only into the skin but also into the muscle area. Animal studies have shown that load transfer to adipose tissue leads to a decrease in adipose tissue volume [38]. Compared to the skin area, where shear forces disrupt neovascularization, grafts in the muscle area experience additional pressure from muscle contractions and stretching forces.

Studies by Hossain et al. demonstrated that compression force inhibits adipogenesis in SGBS cells by suppressing the expression of PPARγ2 and C/EBPα through a COX‐2–dependent mechanism [39]. Tanabe et al. reported that mechanical stretching force inhibits adipogenesis in 3T3‐L1 cells by reducing PPARγ2 expression, mediated by activation of the ERK/MAPK system [40].

Building upon these findings, Shi et al. confirmed that the use of BTX‐A to block nerve transmission and paralyze muscle movement significantly reduces mechanical stimulation to the fat graft and enhances graft retention [36].

Furthermore, Shi et al. conducted experiments injecting BTX‐A mixed with fat grafts intramuscularly. Histological analysis revealed that the adipocytes within the mixed grafts exhibited better integrity compared to the control group. The levels of ADSCs and neovascularization were also increased compared to the control group. These findings suggest that BTX‐A effectively enhances graft retention by inhibiting mechanical stimulation of the graft by the muscle.

We propose that this mechanism is particularly suitable for craniofacial cosmetic treatments, where the movement of facial muscles may inhibit or result in the absorption of grafted adipose tissue during its survival. However, it is important to note that this approach may lead to temporary facial paralysis and a decrease in facial expressions. To mitigate these effects, it is recommended to use micro‐units of BTX‐A for muscle injection in the filling area, reducing the likelihood of overall facial expression disorders.

### 3.2 BTX‐A Improves Graft Preservation by Promoting Angiogenesis

In addition to its well‐known ability to inhibit neurotransmitter transmission and paralyze muscles, BTX‐A has also been found to have a previously unrecognized capacity to promote angiogenesis. Since 1989, Smahel has emphasized the importance of revascularization from adjacent areas in improving the survival rate of adipose tissue compared to other theories of adipose survival [41].

Therefore, it is postulated that the optimal method for preserving transplanted adipose tissue may involve directly promoting vascular regeneration or stimulating stem cell differentiation towards angiogenesis. Recent studies have suggested that BTX‐A may play a role in promoting angiogenesis, leading to enhanced graft survival rates. Baek's team conducted an experiment in 2011, combining BTX‐A with adipose tissue and transplanting it to an injection site. The grafted fat on the BTX‐A side demonstrated statistically significant differences in weight, volume, and integrity compared to the control group after 9 weeks. The BTX‐A group exhibited more intact grafted fat, fewer fat vacuoles, and increased microvasculature around the graft. This experiment provided evidence that combining BTX‐A with adipose tissue can improve retention rates, although the precise mechanism for this increase remains unclear. We propose that the release of neurotransmitters through BTX‐A creates a relatively stable environment for ADSCs, promoting their differentiation towards neovascularization. This, in turn, increases the volume retention of autologous fat grafts in immobilized muscle, stimulates ADSC proliferation and differentiation, and enhances the density of newly formed vessels, ultimately resulting in improved fat retention.

Baek's experiment opened the door for further investigation, offering new insights into the diverse aspects of BTX‐A, a substance commonly recognized for blocking neuromediator transmission [42]. Therefore, BTX‐A can serve as a beneficial adjuvant for adipose tissue grafting [42]. Additionally, the effect of BTX‐A on angiogenesis can vary depending on the concentration, as demonstrated by the adjustment of BTX‐A concentration by Tang et al. Their study with SD rats found that an 8 × 10^−2^ U/mL concentration of BTX‐A was most effective in stimulating ADSC proliferation and differentiation, leading to increased induced revascularization as evidenced by increased VEGF and CD31 levels [18].

Further experiments by Yoon et al. in 2021 using a rabbit animal model confirmed the potential of BTX‐A to enhance adipose angiogenesis [21]. The expression of CD31 increased, and TNF‐α levels were reduced in adipose tissue mixed with BTX‐A, reinforcing the results indicating improved preservation rates of grafted fat in autologous fat transfer.

**FIGURE 2:**
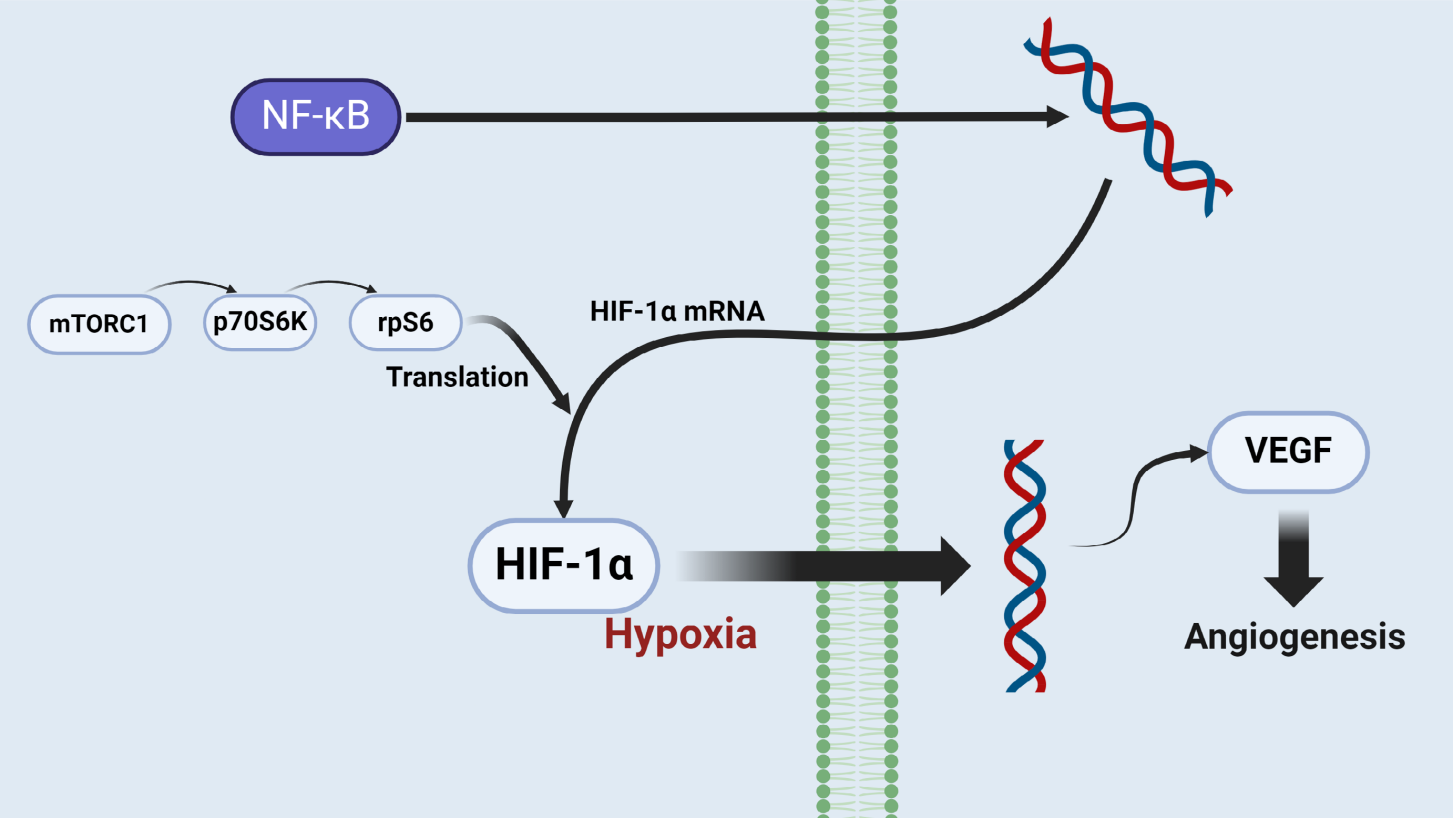
Possible pathways of BTX‐A to enhance angiogenesis.

Researchers are also hypothesizing the underlying mechanism of BTX‐A in angiogenesis. Kim et al. speculated about BTX‐A's molecular mechanism in a rat model in 2015 [43]. Their findings supported the theory that BTX‐A promotes angiogenesis by upregulating the expression of HIF1α and VEGF, possibly through the HIF1α/VEGF signaling pathway. They also discovered an overexpression of mTOR in the BTX‐A group, which may lead to increased HIF1α and VEGF expression [44]. These findings suggest that BTX‐A may promote angiogenesis via the mTOR/HIF1α/VEGF pathway. Another hypothesis suggests that BTX‐A may stimulate NF‐κB in well‐perfused tissues, promoting angiogenesis through the NF‐κB/HIF1α/VEGF pathway (Figure 2). However, considering the relatively low blood perfusion in adipose tissue compared to skin flaps, it is unlikely that adipose tissue promotes angiogenesis via the NF‐κB/HIF1α/VEGF pathway. We hypothesize that BTX‐A promotes the angiogenic capacity of endothelial cells by upregulating the expression of mTOR in hypoxic conditions, thereby enhancing the survival rate of transplanted adipose tissue. Experimental results indicate that under hypoxic conditions, BTX‐A significantly upregulates the expression of mTOR. As a key signaling protein, mTOR activates downstream pathways involving HIF‐1α and VEGF, enhancing the proliferation and migration capabilities of endothelial cells and promoting the formation of new blood vessels. These newly formed blood vessels provide the necessary blood supply to the transplanted adipose tissue, significantly improving its survival rate. In conclusion, Kim's study demonstrates that BTX‐A promotes angiogenesis through the mTOR/HIF‐1α/VEGF pathway under hypoxic conditions, providing an effective mechanism for enhancing the survival rate of transplanted adipose tissue. This finding not only reveals the potential role of BTX‐A in angiogenesis but also offers a new theoretical basis for improving the survival rate of transplanted tissues in clinical applications.

### 3.3 Potential of Using BTX‐A in Autologous Fat Grafting

**FIGURE 3:**
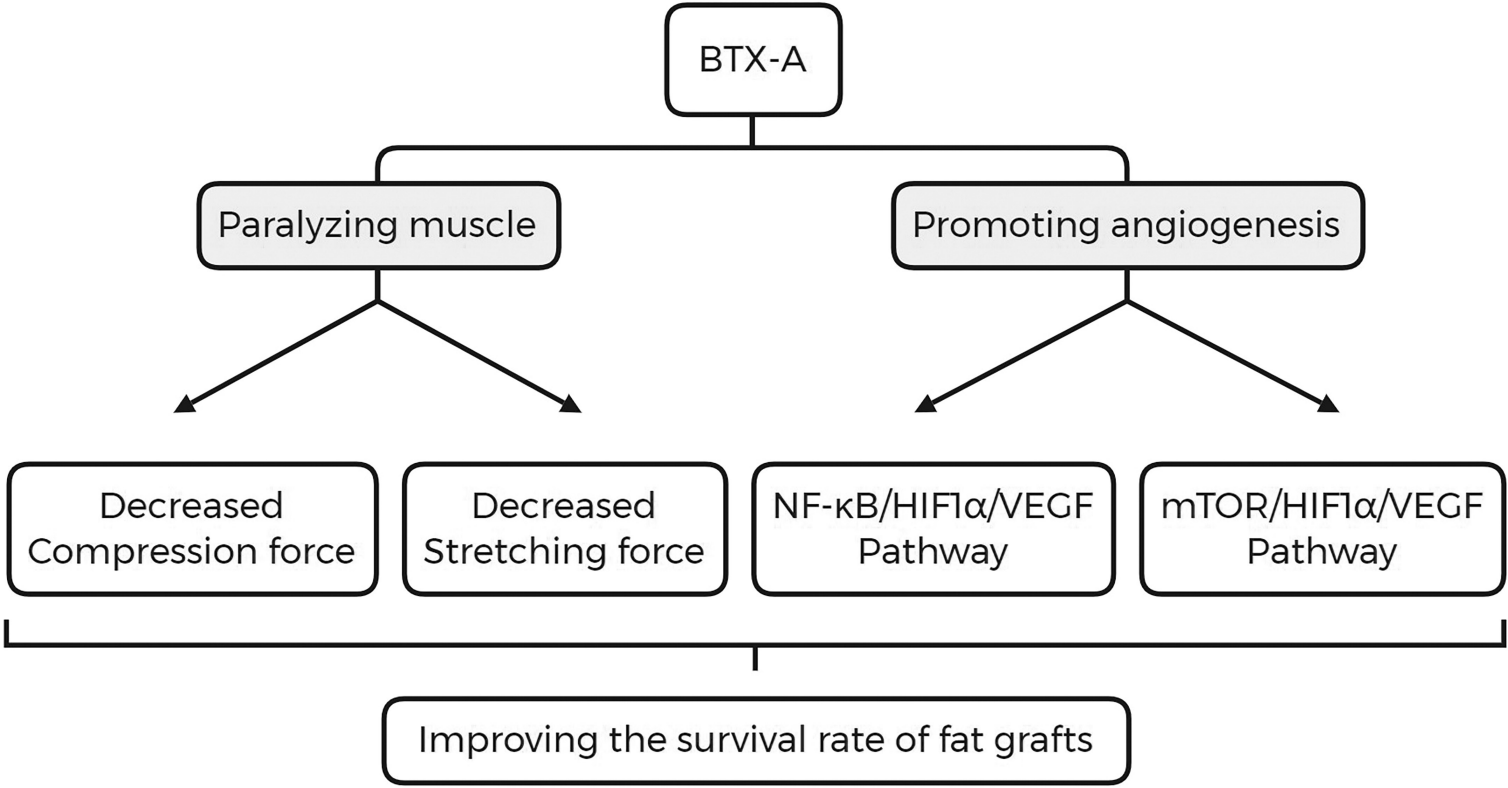
The way to enhance fat survival rate by BTX‐A.

Extensive research has demonstrated the potential of BTX‐A in enhancing fat survival in autologous fat grafting through muscle paralysis and angiogenesis promotion (Figure 3). BTX‐A offers a time‐efficient and convenient approach to improve fat preservation without significantly prolonging surgical procedures. It is particularly effective in injection sites requiring muscle immobilization, such as chin augmentation with undersized mental protuberance. However, the combination of BTX‐A and autologous fat grafts carries risks, particularly regarding dosage control, and is not suitable for multisite multidose facial injections due to potential facial muscle paralysis. Surgeons must exercise caution and remain vigilant about potential complications.

In summary, BTX‐A can increase the retention of autologous fat grafts by immobilizing muscles, promoting vascularization, and facilitating ADSC differentiation. While these outcomes have primarily been observed in small animal models, further research is needed to determine if similar results can be achieved in humans. These findings hold great promise, and future trials are anticipated to explore its full potential.

## 4 Conclusion

Recent studies, conducted by Jung in 2009, Baek in 2012, Tang in 2017, Shi in 2019, Wu in 2020, and Yoon in 2021, have demonstrated the potential benefits of combining BTX‐A with adipose tissue in fat grafting procedures. These findings suggest that the direct promotion of angiogenesis and muscle paralysis contribute to improved fat retention, although the precise mechanism behind these effects requires further investigation. To validate the efficacy of this combination therapy, additional clinical reports and trials are necessary. Surgeons are encouraged to explore this approach within ethical boundaries to confirm its effectiveness and explore its potential applications. While the current evidence is derived from animal experiments, it provides optimism regarding the use of BTX‐A as an adjuvant in autologous fat grafting for enhanced long‐term outcomes. Moving forward, it is imperative to establish standardized protocols and reach a consensus in this field, which will enhance reproducibility and optimize clinical results.

Despite the promising preclinical research demonstrating the potential of BTX‐A in enhancing the survival rate of autologous fat grafting, these findings are primarily based on small animal models. To validate these results in humans, future studies must conduct large‐scale clinical trials. These trials will help ascertain the efficacy and safety of BTX‐A in various clinical scenarios and provide more reliable data to support its application in plastic and regenerative medicine.

Therefore, emphasizing the importance of translational research, translating the current preclinical findings into human clinical trials is crucial. This step will not only confirm the clinical efficacy of BTX‐A but also evaluate its long‐term safety and potential side effects. Existing studies indicate that BTX‐A may cause mild and temporary side effects, such as injection site pain and minor muscle weakness. However, some research also highlights dose‐dependent risks, where excessive doses could lead to muscle paralysis and other serious complications.

Therefore, future research should focus on the following:

1. *Optimizing dosage and injection sites:* Determining the most effective and safest BTX‐A dosage and injection sites to maximize fat graft survival while minimizing side effects.
2. *Long‐term follow‐up:* Conducting long‐term follow‐up studies to assess the enduring effects and safety of BTX‐A in autologous fat grafting.
3. *Multicenter clinical trials:* Performing multicenter clinical trials in diverse clinical settings to verify the generalizability and reliability of the findings.

In summary, while BTX‐A shows significant promise in the field of autologous fat grafting, its application must be substantiated through rigorous human clinical trials. These studies will provide a solid scientific basis for the clinical use of BTX‐A and help optimize the outcomes of autologous fat grafting procedures. Establishing standardized protocols and achieving a consensus in this field will be crucial for enhancing reproducibility and optimizing clinical results.

## Conflicts of Interest

The authors declare no conflicts of interest.

## Data Availability

Data sharing not applicable to this article as no datasets were generated or analysed during the current study.
